# 

**Published:** 1839-01

**Authors:** 


### BRITISH AND FOREIGN MEDICAL REVIEW

OR

### QUARTERLY JOURNAL

OF

### PRACTICAL MEDICINE AND SURGERY

---

EDITED BY

JOHN FORBES M.D. F.R.S.

AND

JOHN CONOLLY M.D.

EDITORS OF THE CYCLOPÆDIA OF PRACTICAL MEDICINE

---

VOL. VII.

OCTOBER—APRIL 1839

S.G.O.L. INDEXED

Surgeon Genl's Office LIBRARY 34530 Washington D.C.

LONDON

JOHN CHURCHILL PRINCES STREET SOHO

MDCCCXXXIX.

W
1
BR34

LONDON:

PRINTED BY C. ADLARD, BARTHOLOMEW CLOSE.

### BOOKS RECEIVED FOR REVIEW.

ENGLISH.

1. Guy's Hospital Reports. No. VII. October, 1838.—pp. 190.

2. The Organ of Hearing. By T. Wharton Jones, Esq. (From the Cyclopædia of Anatomy and Physiology.)—Royal 8vo. pp. 38.

3. Report on the Malignant Fever called the Pali Plague, which has prevailed in some parts of Rajpootana, since the month of July, 1836. Prepared and published by order of the government of India. By James Ranken, M.D., Secretary to the Bengal Medical Board.—Calcutta, 1838. 8vo. pp. 232.

4. Practical Surgery; with 130 engravings on wood. By Robert Liston. Second Edition.—London, 1838. 8vo. pp. 529. 21s.

5. The Principles of Surgery. By John Burns, M.D. F.R.S., Regius Professor of Surgery in the University of Glasgow.—London, 1838. Two Vols. 8vo. pp. 554-535. 24s.

6. The Surgical Anatomy of the Perinæum. By Thomas Morton, formerly one of the House Surgeons of the University College Hospital. Illustrated with Lithographic Plates, and Wood Engravings.—London, 1838. Royal 8vo. pp. 80. 7s. 6d.

7. Lectures on the Physiology and Diseases of the Chest; including the Principles of Physical and General Diagnosis and their Application to Practice. By C. J. B. Williams, M.D. F.R.S.—London, 1838. 8vo. pp. 204. (From the Med. Gazette.)

8. Practical and Surgical Anatomy. By W. J. Erasmus Wilson, Lecturer on Practical and Surgical Anatomy and Physiology. Illustrated with 50 Engravings on Wood.—London, 1838. 8vo. pp. 492. 10s. 6d.

9. Diet and Regimen, physical, intellectual, and moral, as means in the prevention and cure of disease. By Robert Dick, M.D.—Glasgow, 1838. 8vo. pp. 386.

10. Observations on the Oriental Plague, and on Quarantine as a means of arresting its Progress; addressed to the British Association of Science. By John Bowring.—Edinburgh, 1838. 8vo. pp. 45. 1s.

11. Observations on the Anatomy, Habits, and Economy of Athalia Centifoliæ, the Saw-fly of the Turnip. The Prize Essay of the Entomological Society for 1837. With a Plate. By George Newport, Esq.—London, 1838. 8vo. pp. 32. 1s.

12. Practical Observations on the Causes and Treatment of Curvature of the Spine; with hygienic Directions for the Physical Culture of Youth, as a Means of preventing the Disease. By Samuel Hare, Surgeon.—London and Leeds, 1838. Royal 8vo. pp. 151; with Ten Plates. 10s.

13. Outlines of Military Surgery. By Sir George Ballingall, M.D., Regius Professor of Military Surgery in the University of Edinburgh, &c.—Edinburgh, 1838. 8vo. pp. 542. Second Edition. 14s.

14. On the Reform of our Medical Corporations. By a Medical Practitioner at the West End of the Town.—London, 1838. 8vo. pp. 52. 1s.

15. An Account of the Proceedings of the Sixth Anniversary Meeting of the Provincial Medical and Surgical Association, held at Bath, July 1838.—Worcester, 1838. 8vo. pp. 112.

16. On the Objects and mutual Relations of the Medical Sciences; an introductory Address delivered at the Middlesex Hospital School of Medicine, October 2, 1838. By F. T. Leighton, M.D. &c.—London, 1838. 8vo. pp. 51.

17. Remarks on the Poor-law Amendment Act, with reference to Pauper medical Attendance and medical Clubs. By Ed. Copeman, M.R.C.S.—Norwich, 1838. 8vo. pp. 18.

18. The Elements of Materia Medica; comprehending the natural History, Preparation, Properties, Composition, Effects and Uses of Medicines. Part I. By

J. Pereira, F.R.S. &c. — London, 1839. 8vo. pp. 559. 16s.

19. Medical Report of the House of Recovery and Fever Hospital, Dublin, for the year 1836.— Dublin, 1837. 8vo. pp. 39.

20. An Essay on Food, in which the received Doctrine of modern Physiologists respecting the waste of the Body is exploded, &c. &c. By N. Grisenthwaite.—London, 1838. 8vo. pp. 119. 4s.

21. An Account of some new Instruments for tying Polypi of the Uterus, Nose, Ear, and enlarged Tonsils; with Cases. By W. Beaumont, Surgeon to the Islington Dispensary.—London, 1838. 4to. pp. 35; Three Plates. 5s.

22. A Lecture introductory to the Business of the original School of Medicine, Peter-street. By G. T. Hayden, Lecturer on Anatomy and Surgery.—Dublin, 1838. 8vo. pp. 15. 6d.

23. Cyclopædia of Anatomy. Part XV. Hearing (Organ of) to Heat (Animal).

24. Manual of descriptive and pathological Anatomy. By J. F. Meckel. Translated from the French of A. J. L. Jourdan and G. Breschet; with Notes. By A. S. Doane, M.D. and others. Two vols.—London, 1838. 8vo. pp. 571, 650.

25. The Philosophy of Disease; or, a Popular Outline of the Principles of Medical Science: comprising a brief Exposition of the Laws of inflammatory Action. By J. B. Harrison, Surgeon. — London, 1838. 8vo. pp. 152. 4s. 6d.

26. The Medical Portrait Gallery. By T. J. Pettigrew, F.R.S. Part X., containing Mr. Lawrence, Malpighi, Sir John Pringle, Dr. Clutterbuck. 3s.

FOREIGN.

1. Sydenham's Antheil an der Uneinigkeit unserer Lehre über die Gicht. Von Dr. C. J. Heidler.—Berlin, 1838. 8vo. pp. 35.

2. Zur Jubel-Feier des Professor emeritus Dr. Johann Busch in St. Petersburg, am 26sten Mai, 1838.—4to. pp. 32.

3. De Puris natura atque formatione. Disquisitio Physiologica, Auctore Dre. H. Wood.—Berolini, 1839. 4to. pp. 47.

4. Die Achsendrehung des Auges. Von Dr. Alexander Hueck. — Dorpat, 1838. 4to. pp. 35.

5. Die Pathologie und Diagnose der Krankheiten der Brust, ins Besondere erläutert durch eine rationelle Erklärung ihrer physikalischen Zeichen, &c. Von C. J. B. Williams, M.D. &c. Aus dem Englischen ubersetzt und herausgegeben. Von Dr. H. Velten. Zweite deutsche Auflage.—Bonn, 1838. 8vo. pp. 244.

6. Die Chirurgische Muskellehre in Abbildungen. Von Dr. G. B. Günther, Professor der Chirurgie in Kiel.—Hamburg, 1838. 4to.

7. Dissertatio de Anastomosi Jacobsonii et Ganglio Arnoldi. Auctore Hen. Car. Bang Bendz, Med. and Chir. Candidato.—Hauniæ, 1833. 4to. pp. 53. Tab. VIII.

8. De Thalamo et Origine Nervi Optici in Homine et Animalis Vertebratis. Auctore S. A. W. Stein, Chir. and Med. Candidato.—Hauniæ, 1834. 4to. pp. 66. Tab. XII.

9. Abhandlungen aus dem Gebiete der practischen Medicin und Chirurgie. Von Dr. Adolph Leop. Richter, Köngl. Preussischen Regimentsartze, &c.—Berlin, 1832. 8vo. pp. 261.

10. Bemerkungen über den Brand der Kinder. Von Dr. Adolph. L. Richter, &c. —Berlin, 1834. 4to. pp. 22,

11. Theoretisch-praktisches Handbuch der Heilquellenlehre. Von August Vetter, M.D. &c. II. Band.—Berlin, 1838. 8vo. pp. 464-515.

12. Die Kranke Darmschleimhaut in der Asiatischen Cholera mikrokopisch untersucht. Von Dr. Ludwig Boehm. Mit. II. Kupfert.—Berlin, 1838. 8vo. pp. 83.

13. Nova Acta Physico-medica Academiæ Cæsareæ Leopoldino-Carolinæ Naturæ Curiosorum. Tom. XVIII. — Vratislaviæ et Bonæ, 1836-8. 4to. pp. 802.

14. Medicinische Beobachtungen und Bemerkungen. Von J. D. W. Sachse, M.D. &c. Zweiter Band.—Berlin, 1839. 8vo. pp. 359.

15. Morbi Lubeanorum, anno 1828, observati a F. G. Lippich, M.D. &c.—Patavii, 1836. 8vo. pp. 164.

16. Annales Scholæ Medico-clinicæ Patavinæ. Edidit F. G. Lippich, M.D. &c. Annus 1834-5.—Patavii, 1837. 8vo. pp. 183.

17. System der Physiologie für Naturforscher und Aerzte bearbeitet von Dr. C. G. Carus. Erster Theil.—Dresden, 1838. 8vo. pp. 372.

18. Observationes Anatomicæ et Microscopicæ de Systematis Nervosi Structura. Auctore Roberto Remak, M.D.—Berolini, 1838. 4to. pp. 41; cum ii. Tab.

19. Teoria della Flogosi di Giovanni Rasori.—Livorno, 1837. 8vo. pp. 260.

20. Ueber die Ursachen der grossen Sterblichkeit der Kinder in ihrem ersten Lebensjahre. Eine gekrönte Preisschrift von E. F. Frohbeen, M.D.—Dorpat, 1837. 8vo. pp. 130.

21. Handbuch der Physiologie des Menschen fur Vorlesungen. Von Dr. Johannes Müller, &c. II. B., i. und ii. Abtheil.—Coblenz, 1838. 8vo. pp. 502.

